# Supplementary material for: Weight loss effects of non-pharmacological interventions in women with polycystic ovary syndrome: a systematic review and network meta-analysis
Source: PeerJ. 2025 Apr 16;13:e19238. doi: 10.7717/peerj.19238 (PMC12009027; doi:10.7717/peerj.19238)
Supplement: Supplemental Information 1 [file peerj-13-19238-s001.docx]

**The reason for this study:**

Obesity is a primary trigger for polycystic ovary syndrome (PCOS) and serves as its main clinical manifestation. There are various approaches to weight loss in patients with PCOS, yet the most effective means of achieving weight loss in this population remains to be determined by scientific evidence.So we conducted a related mesh meta-analysis to determine the best weight loss intervention.

**What is New**

The results of this study show that tai chi is particularly beneficial for weight loss in PCOS patients, suggesting that simply increasing exercise intensity is not the only way to lose weight. However, more randomized controlled trials are needed to determine the weight loss effects of combining exercise and diet.
