# Supplementary material for: Weight loss effects of non-pharmacological interventions in women with polycystic ovary syndrome: a systematic review and network meta-analysis
Source: PeerJ. 2025 Apr 16;13:e19238. doi: 10.7717/peerj.19238 (PMC12009027; doi:10.7717/peerj.19238)
Supplement: Supplemental Information 3 [file peerj-13-19238-s003.docx]

TableS1. PubMed Search History

| Databases | Query | Results |
| --- | --- | --- |
| PubMed | ((((((((((((((((((((((((((((((((non-pharmacological interventions[Title/Abstract]) OR (non-drug treatment non-medicine treatment[Title/Abstract])) OR (non-pharmaceutical therapy[Title/Abstract])) OR (diet*[Title/Abstract])) OR (nutritional supplement[Title/Abstract])) OR (Dietary Supplements[Title/Abstract])) OR (Nutritional Support[Title/Abstract])) OR (nutrition[Title/Abstract])) OR (exercise*[Title/Abstract])) OR (exercising[Title/Abstract])) OR (sport*[Title/Abstract])) OR (run[Title/Abstract])) OR (swim*[Title/Abstract])) OR (train*[Title/Abstract])) OR (walk*[Title/Abstract])) OR (acupuncture[Title/Abstract])) OR (electroacupuncture[Title/Abstract])) OR (acupuncture points[Title/Abstract])) OR (moxibustion[Title/Abstract])) OR (Tai Chi[Title/Abstract])) OR (cognitive therapy[Title/Abstract])) OR (Psychotherapy[Title/Abstract])) OR (psychosocial[Title/Abstract])) OR (Behavior Therapy[Title/Abstract])) OR (Cognitive Behavioral Therapy[Title/Abstract])) OR (life* style[Title/Abstract])) OR ("Dietary Supplements"[Mesh])) OR ("Diet"[Mesh])) OR ("Sports"[Mesh])) OR ("Acupuncture"[Mesh] OR "Acupuncture Therapy"[Mesh])) OR ("Cognitive Behavioral Therapy"[Mesh])) OR ("Exercise"[Mesh] OR "Exercise Therapy"[Mesh] OR "Resistance Training"[Mesh])) AND (((Polycystic Ovary Syndrome[Title/Abstract]) OR (PCOS[Title/Abstract])) OR ("Polycystic Ovary Syndrome"[Mesh])) | 2812 |
