# Supplementary figures and images for: Weight loss effects of non-pharmacological interventions in women with polycystic ovary syndrome: a systematic review and network meta-analysis
Source: PeerJ. 2025 Apr 16;13:e19238. doi: 10.7717/peerj.19238 (PMC12009027; doi:10.7717/peerj.19238)

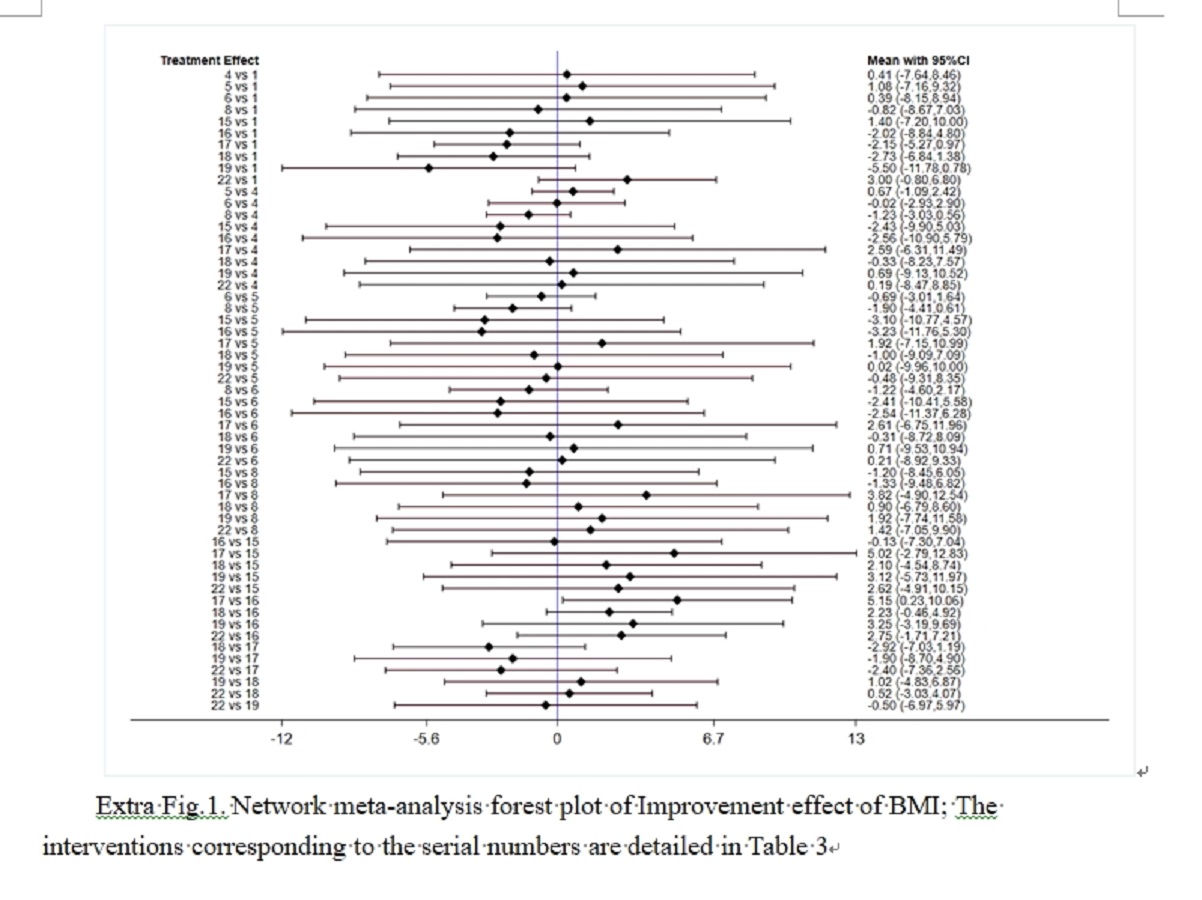

Supplement: Supplemental Information 4 [file peerj-13-19238-s004.jpg]

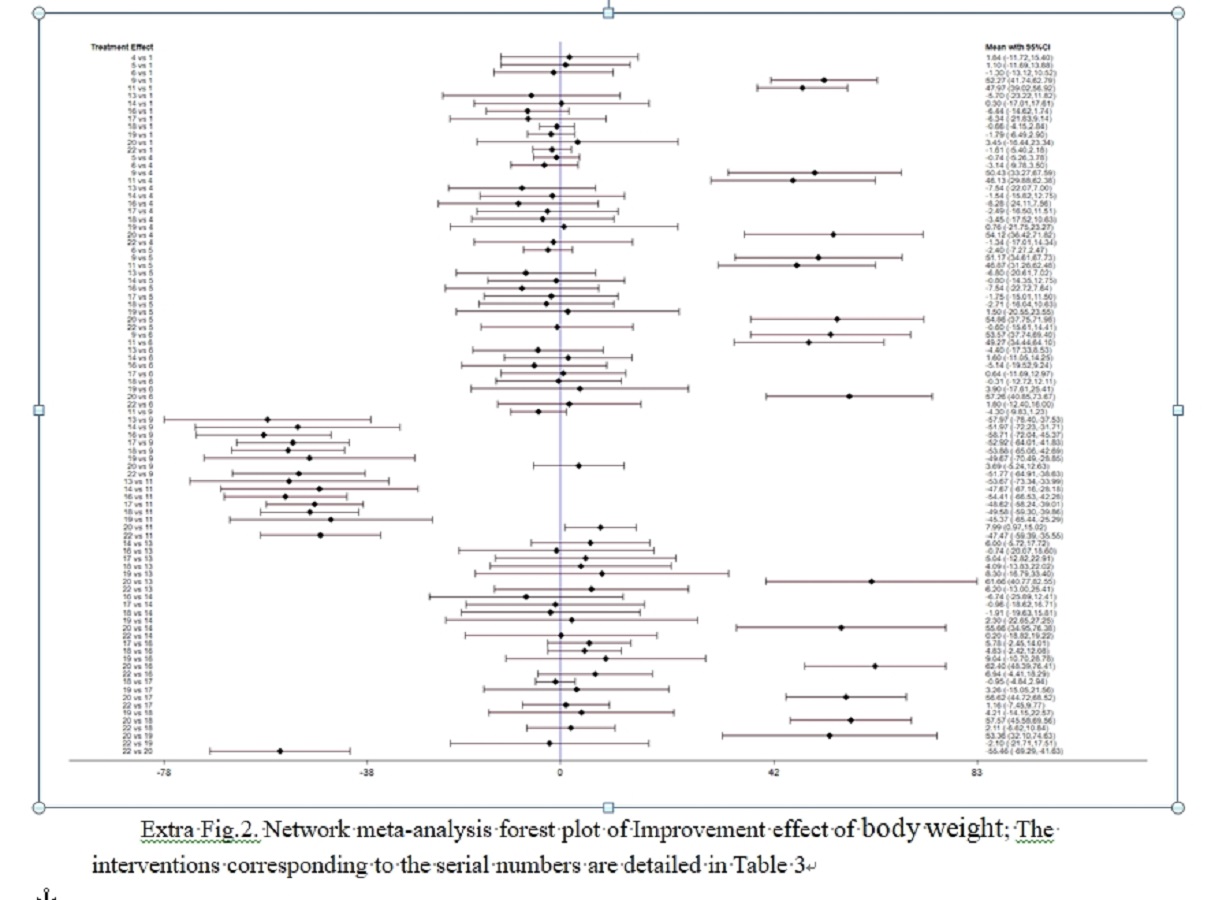

Supplement: Supplemental Information 5 [file peerj-13-19238-s005.jpg]

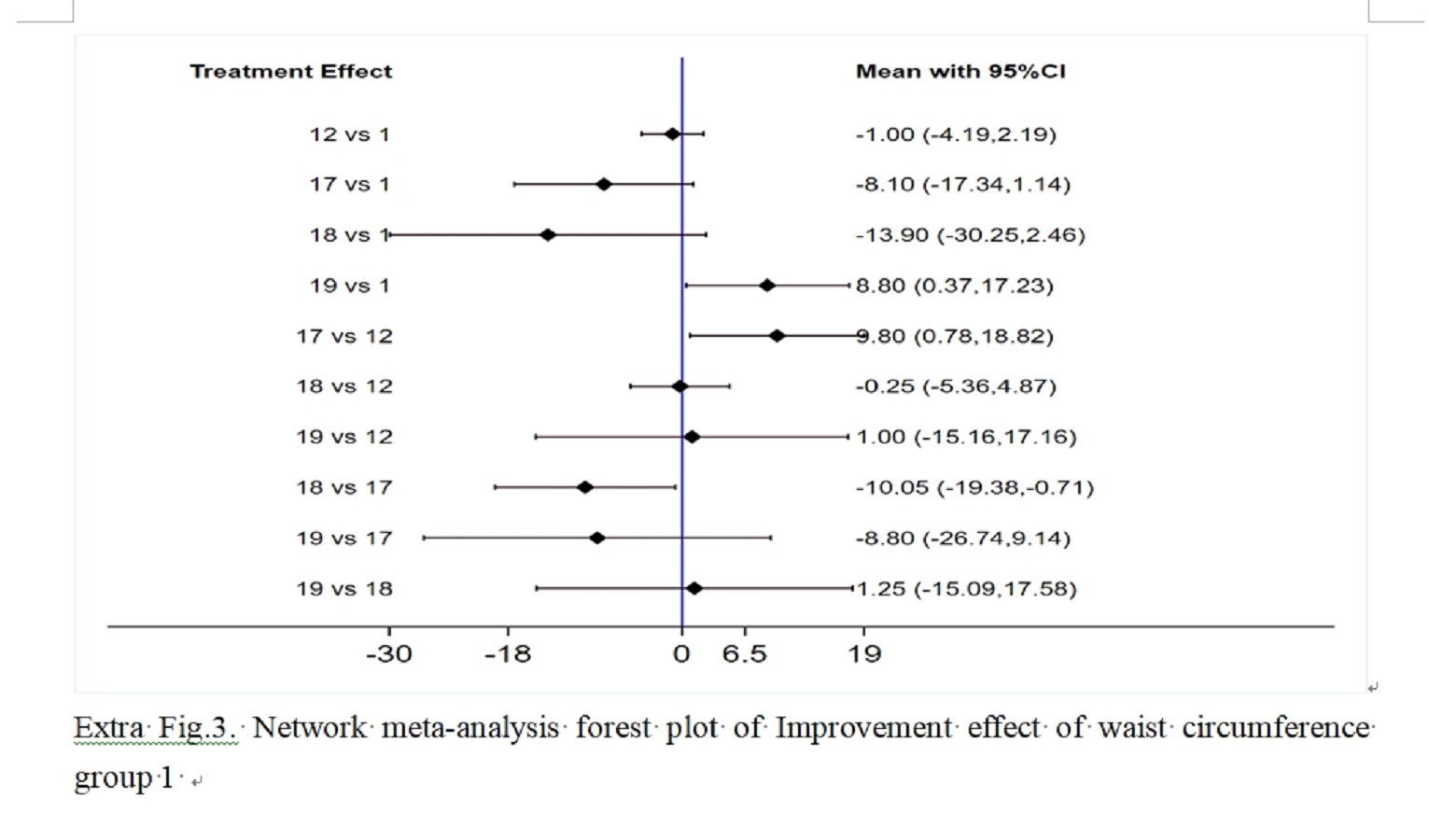

Supplement: Supplemental Information 6 [file peerj-13-19238-s006.jpg]

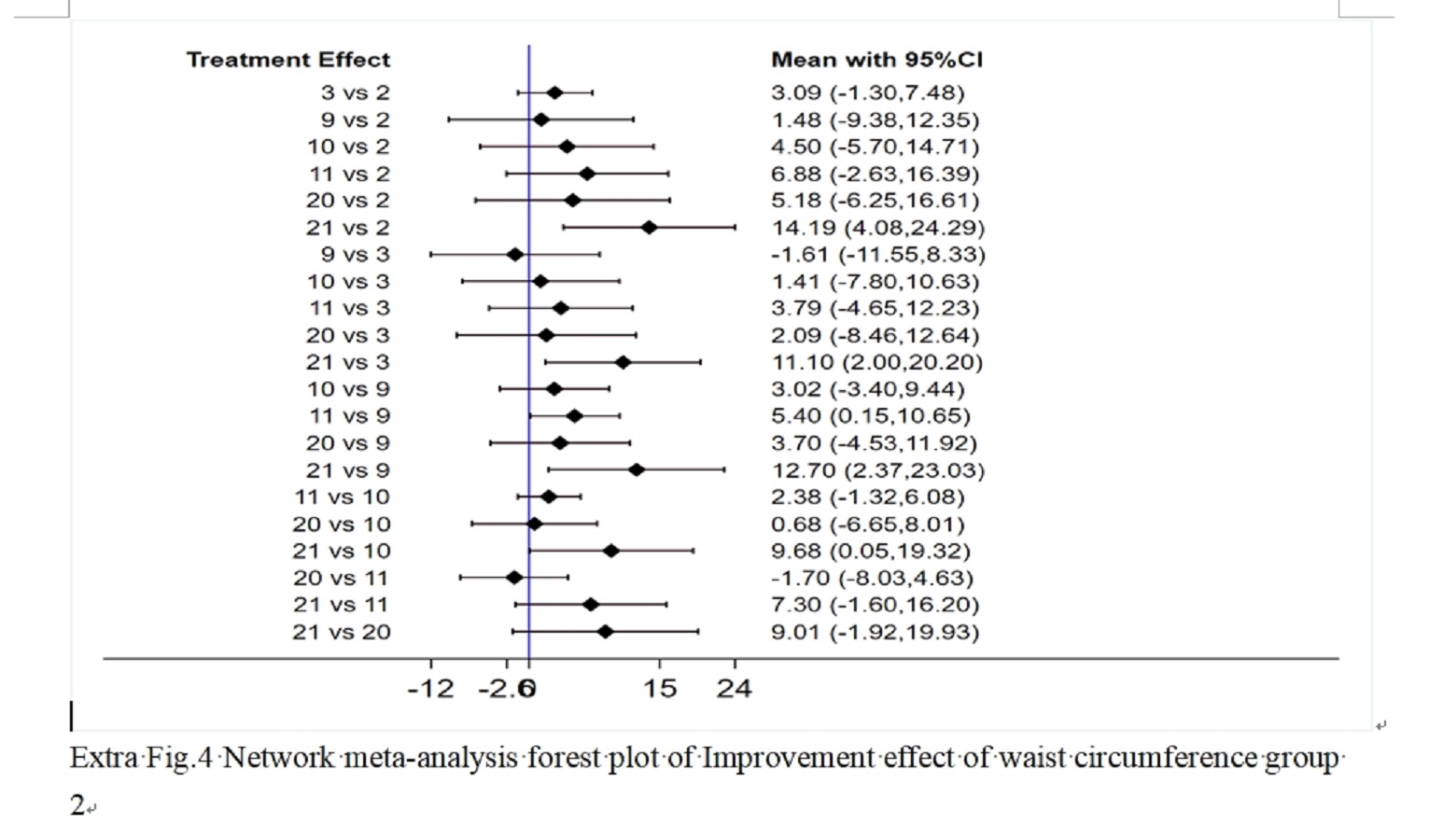

Supplement: Supplemental Information 7 [file peerj-13-19238-s007.jpg]

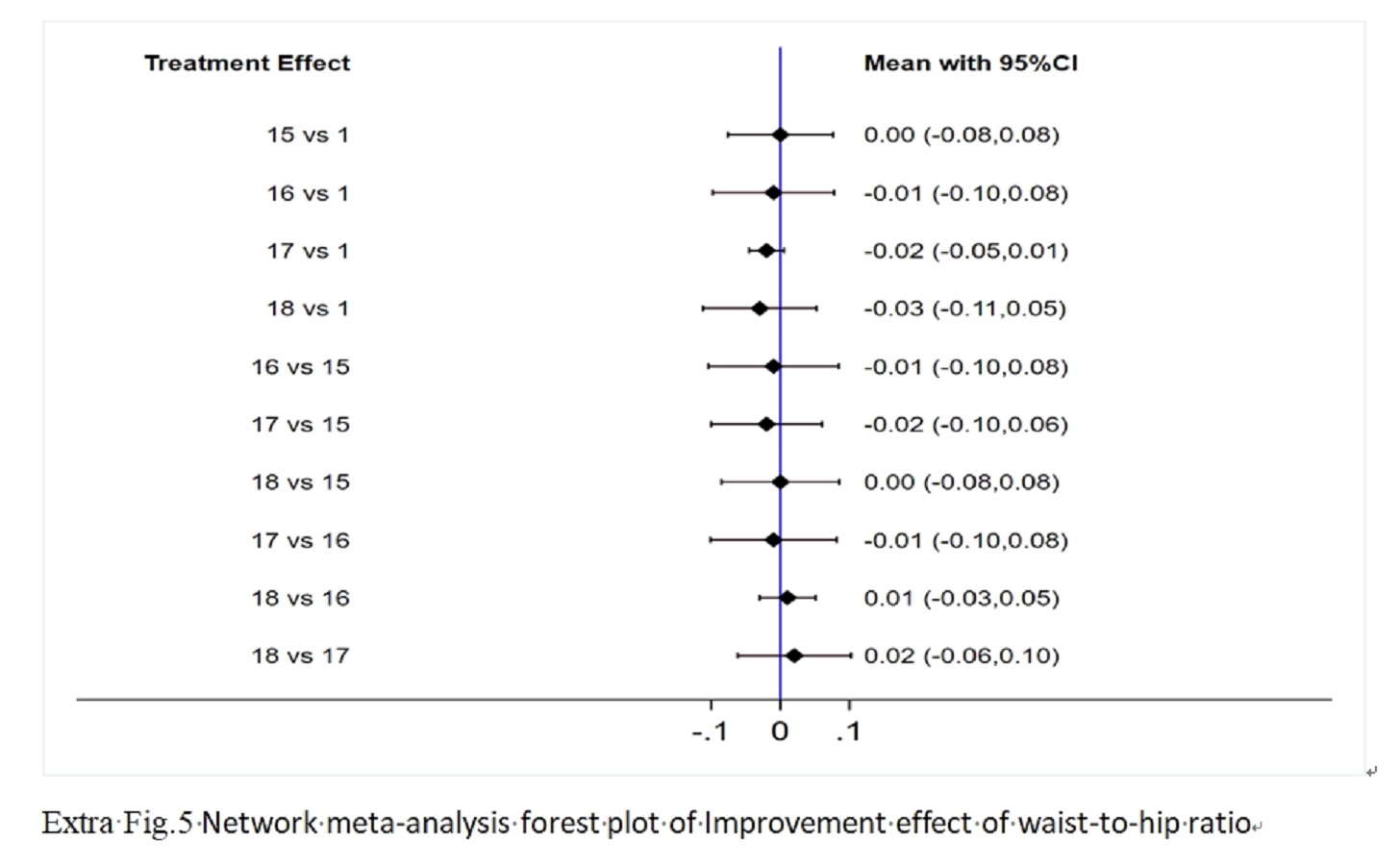

Supplement: Supplemental Information 8 [file peerj-13-19238-s008.jpg]
